# Gender Differences in Article Citations by Authors from American Institutions in Major Radiology Journals

**DOI:** 10.7759/cureus.5313

**Published:** 2019-08-03

**Authors:** Mingqian Huang, Kiyon Naser-Tavakolian, Michael Clifton, Ana M Franceschi, Derek Kim, Jill Z Zhang, Mark Schweitzer

**Affiliations:** 1 Radiology, Stony Brook University Hospital, Stony Brook, USA

**Keywords:** female authorship, gender differences, radiology journals, citations

## Abstract

Objective

To investigate gender difference patterns in article citations, by first and last authors, in four radiology journals.

Materials and methods

Articles by authors published in four major radiology journals from 1984, 1994, 2004, and 2014 were categorized into 12 subspecialties. The number of citations, references used, co-authors, and length of the article (number of pages) were documented. The genders of first/last authors were determined. Data were analyzed using chi-square and logistic regression.

Results

The gender of the first author was determined in 2679 articles and that of the last author in 2717 articles. Over the selected years, 1984 to 2014, female first authorship grew from 13.0% to 31.5% (p<0.001), and female last authorship grew from 9.3% to 22.1% (p<0.001). Primary female authorship papers were cited less often as compared to males (OR 0.9972, 95% CI: 0.9948-0.9996, p=0.021), after adjusting for publication year and subspecialty. Across most subspecialties, female first authorship received fewer citations. In 1984, primary female authorship papers received on average 28.9 citations versus males at 39.1; in 1994, 50.4 versus 60.8; in 2004, 41.5 versus 44.4; and in 2014, 7.0 versus 7.8. The mean difference in the number of citations received by male and female first authors decreased from 10.47±6.09 in 1984 and 9.49±7.12 in 1994 to 1.93±5.63 in 2004 and 0.79±0.39 in 2014. However, there was no statistical difference demonstrated in article citations between male and female last authorship (OR 0.9990, 95% CI: 0.9966-1.0013, p=0.392).

Conclusions

Primary female authorship garnered fewer citations than men, despite the increasing frequency of authorships. However, this differential in the number of citations is narrowing.

## Introduction

In the past several decades, participation in the medical profession has equalized among men and women. Currently, approximately equal numbers of women and men enter and graduate from medical school in the United States [1]. Since 2009, the male to female ratio of students enrolling in U.S. medical schools has been 1.10-1.15. Over the last four years, since 2014, the number of female students entering medical school has steadily increased. In 2018, the ratio of men to women approached 1:1 [2].

Despite this growing trend, radiology remains a specialty that is underrepresented by women [3]. In 1990, women comprised 25.5% of the radiologist workforce while in 2013 [4], the percentage increased to 27% [5]. Women remain underrepresented in all levels of rank within academic radiology, especially senior faculty. Data have shown that women are more likely than men to begin academic careers in medicine after training, yet they lag behind their male counterparts in obtaining senior faculty positions [6]. According to the Association of American Medical Colleges (AAMC) report in 2014, women comprise 19.2% of full professors, 25.6% of associate professors, and 31.3% of assistant professors among American radiology faculty [5].

Academic advancement is mainly dependent on scholarly productivity based on peer-reviewed original research and editorial publications [7]. Recently, considerable research investigating female authorship in many specialties has been conducted in medicine, including internal medicine [8], orthopedic surgery [9], gynecologic oncology [10], and radiation oncology [11]. The gender gap of authorship in major radiology journals has received considerable interest [12-14]. However, to date, no study has objectively reviewed gender differences in the number of citations received by an author in academic radiology.

Here, we first examine the prevalence of female primary and last authors of original research in four high-impact general radiology journals, examining changes over a 30-year time frame. We then emphasized gender differences in citations. The articles were sub-categorized into 12 radiologic subspecialties (organized by the highest impact journal, "Radiology") and compared the citations received by female or male authors in each of the 12 subspecialties. Our goal was to help better understand women's representation among academic radiologists and further promote female leadership.

## Materials and methods

This study is a retrospective bibliometric analysis of gender differences in academic literature in radiology. The study did not constitute human subject research and thus did not require local institutional review board approval.

Inclusion and exclusion criteria

We selected four high-impact radiology journals: Radiology, European Journal of Radiology (EJR), Journal of Computer Assisted Tomography (JCAT), and American Journal of Roentgenology (AJR). To ensure a representative sample of articles published during the last three decades, we selected the years 1984, 1994, 2004, and 2014 spread apart by 10-year intervals. The final year studied was 2014, to allow for a broad timespan of potential citations. Only hypothesis-driven original research articles were included in this study. Book reviews, commentaries, review articles, letters, and quizzes were excluded.

The Web of Science database was utilized, and a filter was applied for the years 1984, 1994, 2004, and 2014 to gather the variables of interest. Initials were only listed for some of the articles from 1984 and 1994 in the Web of Science database. We obtained the first and last author's names from each journal's electronic archives. The first and last authors from American medical institutions were selected for this study, as gender-specific names were more easily identified.

Variables assessed

For each article, we obtained data regarding the name of the article, time of publication (year), names of the first and last authors, total number of co-authors, article length, number of references used, and number of citations received by the time of data collection in March 2017. We did not filter for self-citations, as filtering for each author was not feasible due to the large number of articles that were reviewed. All of the articles were separated into 12 radiology subspecialties by a board-certified musculoskeletal radiologist with seven years of practice, based on the titles of the articles. The 12 subspecialties included breast imaging, cardiothoracic imaging, gastrointestinal imaging, genitourinary imaging, health policy and practice, musculoskeletal imaging, neuroradiology, nuclear medicine, pediatric imaging, technical developments, vascular and interventional, and ultrasound. This follows the categorization method of the academic journal Radiology.

The gender of the first and last authors was assessed by inspection of the first name, as most first names are only associated with a specific gender (e.g., "John" is male and "Jessica" is female). If an author's gender was unclear, we conducted an Internet search using three gender-guessing websites [15-17] to determine the likely gender of the authors. These websites analyze first and middle names to determine the likelihood of their association with a specific gender. When the gender of the author could be determined by at least two of the three websites, then that gender was recorded. If only the initials of the first name were used in the list of the authors, we performed an Internet search using the Google search engine to find the first name and then followed the steps mentioned above.

If the above measures could not successfully determine an author's gender, then attempts were made to gain additional information about the author. Internet searches using the Google search engine were conducted by visiting institutional websites of the author's affiliated institution (some of which would contain photographs). Authors were excluded from the study if any of the above steps could not determine their genders.

All the above data were incorporated into a spreadsheet using Excel (2016, Microsoft Corp., Washington, US). The data analysis was performed using Stata 14.0 (StataCorp LLC, College Station, TX, US). First, a chi-squared test was used to assess the associations between the gender of the first and last authors and the year of publication (which was treated as a categorical variable), the total number of co-authors, number of references used, number of citations, and radiology subspecialties. Multiple logistic regression was then used to further evaluate the independent association between author gender and variables of interest. The overall significance level for the study was set at p < 0.05 using a two-sided paired T-test.

## Results

A total of 2864 articles (1152 from 1984; 672 from 1994; 516 from 2004; and 524 from 2014) was collected from a search through Web of Science of the four journals. A total of 1258 articles were collected from Radiology, 1180 articles were collected from AJR, 255 articles were collected from JCAT, and only 71 articles from EJR, likely due to the lower number of authors from American institutions. Among these, we were able to determine the gender of first authors of 2692/2864 articles (94.0%) and last authors of 2730/2864 articles (95.3%). From 1984 to 2014, female first authorship grew from 13.0% to 31.5% (p<0.001), and female last authorship grew from 9.3% to 22.1% (P<0.001). Table 1 outlines this information.

**Table 1:** Number of articles obtained and organized by first and last author and gender. Number of female first and last authors by decade.

	First Authors (%)	Last Authors (%)
Unknown (excluded)	38	24
Non-identifiable non-US based authors (excluded)	134	110
Male	2149 (79.8)	2353 (86.2)
Female	543 (20.2)	377 (13.8)
	Female first authors (%)	Female last authors (%)
1984	145 (12.9)	105 (9.3)
1994	136 (21.0)	104 (16.0)
2004	119 (25.3)	64 (13.5)
2014	143 (31.6)	104 (21.9)

Table 2 shows the number and percentage of first and last female authors by the various subspecialties. Concerning individual subspecialties, breast imaging had the highest rate of both female first and last authors, at 48.3% and 40.1%, respectively. Technical advancement papers had the fewest female first authors, at 5.5%, and vascular and interventional had the lowest percent of last female author at 9.1%.

**Table 2:** Proportion of female first and last authors by specialty in all articles that were reviewed.

	First authors		Last authors	
	Total	Female (%)	Total	Female (%)
Genitourinary	188	47 (25)	191	27 (14.1)
Health policy and practice	223	34 (15.2)	225	32 (14.2)
Interventional and vascular	354	47 (13.3)	362	33 (9.1)
Musculoskeletal	286	48 (16.8)	292	39 (13.4)
Nuclear	113	18 (15.9)	114	12 (10.5)
Pediatric	160	54 (33.7)	162	34 (21)
Technical advancement	55	3 (5.5)	55	5 (9.1)
Ultrasound	123	39 (31.7)	121	33 (27.3)
Body	416	85 (20.4)	413	38 (9.2)
Cardiothoracic	296	55 (18.6)	307	31 (10.1)
Mammography	143	69 (48.3)	147	59 (40.1)
Neuroradiology	322	41 (12.7)	328	34 (10.4)
Total	2679	540 (20.2)	2,717	377 (13.9)

**Figure 1:**
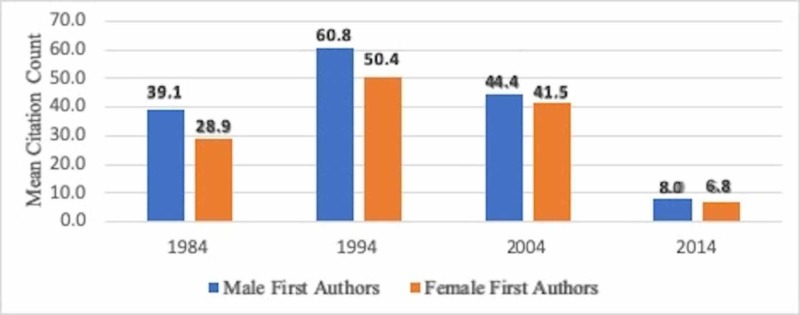
Average citations by male and female first authors.

In 1984, articles by female first authors received an average of 28.9 citations vs. males at 39.1; in 1994, 50.4 vs. 60.8; in 2004, 41.5 vs. 44.4; and in 2014, 6.8 vs. 8.0 (Figure 1). Overall, articles by female first authors were cited less often when compared to their male counterparts (OR 0.9972, 95% CI:0.9948-0.9996, p=0.021), even after adjusting for publication year and subspecialty.

**Figure 2:**
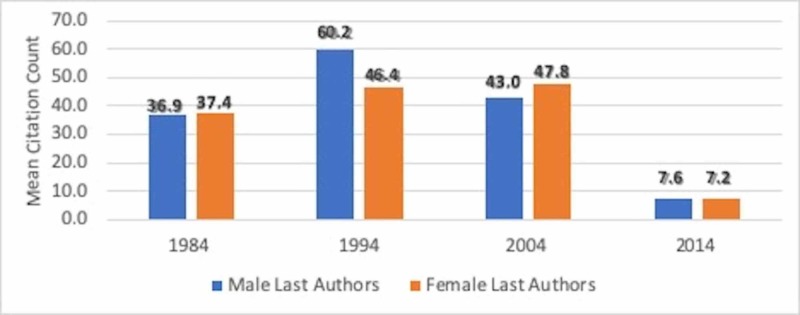
Average citations by male and female last authors.

The mean difference of citation between male and female first authors decreased markedly from 10.47±6.09 in 1984 and 9.49±7.12 in 1994 to 1.93±5.63 in 2004 and 0.79±0.39 in 2014 (Figure 1). No difference in the number of citations received for female or male last authors was found (OR 0.9990, 95% CI:0.9966-1.0013, p=0.392) (Figure 2). Of note, however, in 2004, female last authors had garnered a higher number of citations as compared to men, which again supports a positive trend in citations of female authors.

Additionally, there was no significant difference in the number of references used in articles by the first female or male authors (p=0.25). There was no difference in the length of the article when comparing first female or male authors (p=0.91). Lastly, there was no difference in the number of co-authors per article between first female or male authors (p=0.81).

## Discussion

Our study provides some interesting and challenging data regarding potential gender biases in imaging literature. Notably, the disparity between male and female authorship has decreased over the last three decades, reflecting the changes that have occurred in other medical specialties [9-12]. Additionally, female authors are less prevalent than male authors [12] and their papers are also cited less often.

We chose to look at several variables to explain the discrepancy between male and female authors. The length of papers is correlated with citations [18-20], but the manuscript length did not explain the gender differences we found. Additionally, the number of references was similar between the first author papers by both genders. A paper's content and the strength of the conclusion may have an impact on how often it was cited. This is evidenced by the large number of citations of studies from the 1980s as compared to the past two decades and may be due to the technological advancements that resulted in "landmark" papers that continued to be cited over the course of decades.

It is well-known that specific subspecialties in medicine are cited more frequently [21-22]. Although we found female authorship more common in breast imaging (the only subspecialty in imaging with female authorship as the majority), the subspecialties of the papers did not seem to explain the gender differences in citations. We believe that the reason for increased female authorship in breast imaging is due to the prevalence of female breast imagers as compared to men [23]. Additionally, authors from subspecialties might tend to publish in more subspecialty focused journals, whereas the journals we included in the study cover all subspecialties of radiology. An interesting finding in our results was that there was no significant difference in the number of citations for male or female last authors. However, there was a difference in first authorship between males and females. This may be explained by the increased female senior role in academia over the past four decades.

We also looked at the number of co-authors since there is a good body of literature stating that, overall, women are better team builders [24-25]. However, we found that the number of co-authors was similar between authors of either gender or that this was not associated with changes in the frequency of citations. Additionally, we don't believe this is a significant factor in the difference in the number of citations because larger research groups likely have subdivisions dedicated to various projects. This would subdivide the authorship based on the various subgroups, which would limit the number of co-authors.

There are many decisions involved in an author choosing to include a piece of literature for publication. As such, an author may be more likely to include an article that supports their particular paper rather than selecting a reference from a high-impact paper, which may not effectively strengthen their claim. Furthermore, authors, as a whole, are more likely to cite themselves in subsequent manuscripts. Self-citation likely plays a significant role in the number of citations an author receives over their lifetime. Whether men are more likely to self-cite than women is also something that can be further studied.

Female leadership in radiology has lagged behind their male counterparts'. In particular, seniority, international exposure, and industry contacts have been shown to have a higher association with increased paper citations [26]. This appears to be a significant factor for the differences in the number of citations received, as seen in 2004 when female senior authors garnered a higher number of citations as compared to men.

On the other hand, it's possible that one does not look at the gender of the author when citing their work. Authors may not consciously be aware of the gender of the author they're citing until they include the paper in their references. At that point, it is unlikely that they change their citation based on this finding. As such, we do not believe the authors choose or exclude literature based on the gender of the author. However, this could be a further point of study to determine if authors consciously or subconsciously make a note of the gender of the author they are about to cite. This, however, can only be accomplished by interviewing various authors to identify how they choose their references. 

Limitations

The limitations of this study include that our data sampled only four general radiology journals, which limits the generalizability of our research to other journals, especially subspecialized radiology journals. Additionally, we did not control for the journal in which the article was published. As such, it is possible that a more prestigious journal had articles with higher citation rates. With regards to our methodology, we did not account for self-citations because of the large sample size of papers and authors we identified. We did not find it feasible in our study to determine self-citation rates for each author and, as such, this is a limitation. As previously stated, the gender of authors was determined by an inspection of their first name, which has been used in several articles published on gender trends. However, this is also a limitation, as there may be false identifications using this method. Additionally, we selected only one year per decade due to the extraordinary number of papers that needed to be scientifically abstracted. Of note, a significant number of articles came from 1984. One explanation for this would be that in the 1980s, numerous advances in imaging occurred, including the development of magnetic resonance imaging, computed radiography, and Doppler ultrasound imaging, all of which would provide new avenues for publications [27].

Additionally, multiple regression analyses investigating the influences of potential factors, including gender, on the number of citations might provide further information. Future identification of other factors that may play a role as barriers or enhancers of narrowing the gender gap would provide more insight into this subject.

## Conclusions

Overall, female first authorship garnered fewer citations than men, despite increasing authorships over the past 30 years, but this differential is narrowing. A positive trend is noted in female first and last authorship over the past four decades and this trend will likely continue. By recognizing and understanding citation patterns by gender, female radiologists may further benefit in increasing their academic productivity.
